# Interictal Epileptiform Discharges Might Be More Likely During Particular Phases of Brain Activity

**DOI:** 10.3389/fneur.2015.00253

**Published:** 2015-12-04

**Authors:** David F. Abbott

**Affiliations:** ^1^The Florey Institute of Neuroscience and Mental Health, Austin Hospital, Melbourne, VIC, Australia; ^2^The University of Melbourne, Melbourne, VIC, Australia

**Keywords:** epilepsy, functional magnetic resonance imaging, fMRI, functional connectivity, default mode network, dynamic, temporal lobe epilepsy, genetic generalized epilepsy

Many epilepsy patients exhibit intermittent abnormal brain activity, evident as abnormal electrical events on electroencephalography (EEG), at times when they are not having a seizure. Despite the ease with which these so-called interictal epileptiform discharges (IEDs) can be detected, and a reasonable understanding of the physiological processes occurring during the IED (1), the effect of IEDs on brain function and the nature of their association with seizures remains unclear.

Several recent studies of the potential effects of IEDs on brain function have examined activity within the default mode network [DMN; (2)], a major distributed functional network of the healthy brain typically associated with introspective tasks such as autobiographical memories, internal rumination, or random episodic silent thinking [REST; (3)]. Major nodes of the DMN include posterior cingulate/retrosplenial cortex, medial prefrontal cortex, and the inferior parietal lobule (4). It has been postulated that abnormal activity in the DMN may be a causative factor in loss of awareness during seizures (5). Studies of IEDs have demonstrated a temporal association between the discharges and activity changes in nodes of the DMN. For example, deactivation of DMN is observed in several generalized and focal epilepsies (6–10), and activation in DMN is associated with some IED types (11). However, a causal link has not been definitively established: IEDs might cause DMN changes, or there may be certain, perhaps normal, functional states of the brain that are more permissive than others to the occurrence of epileptiform activity (12–15).

Lopes et al. have now addressed this issue by measuring the intrinsic functional connectivity of the DMN in patients immediately before, during and after epileptiform activity, and comparing the connectivity in these periods with a baseline well away from the epileptiform activity (16). They utilized sliding-window dynamic functional connectivity, a multivariate approach that assesses correlations in activity between brain regions over relatively short timespans (typically of the order of tens of seconds) (17). Lopes et al. studied both idiopathic generalized epilepsy (IGE) [i.e., genetic generalized epilepsy (GGE) (18)] and temporal lobe epilepsy (TLE).

An interesting observation of this study is an overall increase in intrinsic connectivity in portions of the DMN several seconds prior to the onset of IEDs in both GGE and TLE. That this increase is observed prior to the IED suggests it is not the IED causing the change. The authors suggest that the “DMN connectivity may facilitate IED generation.” The fact that the increase in DMN activity in GGE patients occurred bilaterally, while in TLE patients, it occurred mainly in nodes ipsilateral to the affected temporal lobe, is consistent with the authors’ hypothesis. However, while the hypothesis is plausible, there exist other possible explanations. For example, EEG spikes become visible only when a large area of cortex is synchronously active (19). Abnormal activity not visible on EEG may therefore be present prior to the IED, building to a stage that ultimately culminates in the observed IED. In the present case, pre-IED abnormal activity might have caused both the observed DMN connectivity changes and the IED. Nevertheless, it seems reasonable to conclude at least that the IEDs observed on EEG did not cause the preceding DMN changes in the studied patients.

Although intriguing, results at this stage are from small subject numbers (six GGE and six TLE patients), due largely to the difficulty in collecting suitable data (studies with sufficient interictal events spaced far enough apart). The results can therefore only be considered pilot in nature. The study points the way forward for larger studies to investigate these issues. One possibility in this regard is greater sharing of data across centers. Lopes et al. themselves obtained data across two centers; sharing of data across a greater number of centers may yield a more substantial cohort.

Independently of Lopes et al., Faizo et al. recently studied functional connectivity between hippocampi immediately prior to IEDs in a group of 15 mesial TLE patients (20). They observed functional connectivity decrease prior to the IED onset. Taken together with the observations of Lopes et al., this suggests there may be systematic complex alterations in functional connectivity, including both increases and decreases, between different brain nodes prior to IEDs.

The work of Lopes et al. and others demonstrate the value of neuroimaging studies of the peri-event dynamics of IEDs, justifying increased effort in this research domain. Understanding the nature of interactions between “normal” and “abnormal” brain activity may provide new clues as to the nature of various epilepsy phenotypes, and potentially novel avenues for treatment. In addition to larger studies of GGE and TLE, it would be useful if future studies examined additional brain networks, and other epilepsy syndromes.

## Conflict of Interest Statement

The author declares that the research was conducted in the absence of any commercial or financial relationships that could be construed as a potential conflict of interest.
